# Suppression of IL-23-mediated psoriasis-like inflammation by regulatory B cells

**DOI:** 10.1038/s41598-021-81588-8

**Published:** 2021-01-22

**Authors:** Kie Mizumaki, Motoki Horii, Miyu Kano, Akito Komuro, Takashi Matsushita

**Affiliations:** 1 Department of Dermatology, Faculty of Medicine, Institute of Medical, Pharmaceutical and Health Sciences, Kanazawa University, Kanazawa, 920-8641 Japan; 2 Department of Plastic Surgery, Kanazawa University Hospital, Kanazawa, 920-8641 Japan

**Keywords:** B cells, Chronic inflammation, Skin diseases

## Abstract

Psoriasis is an inflammatory cutaneous disease mediated by T-cell dependent immune responses; however, B cells are also considered to play an important role its development. Regulatory B cells (Bregs) regulate immune responses negatively through interleukin-10 (IL-10) production. This study aimed to investigate the role of Bregs in IL-23-mediated psoriasis-like inflammation in mice. Psoriasis-like inflammation was induced in B cell-specific phosphatase and tensin homolog (PTEN)-deficient mice, in which Bregs were significantly expanded, and in their controls, by intradermal injection of 20 μL phosphate-buffered saline (PBS) containing 0.5 μg rmIL-23 into one ear, every other day for 16 days. IL-23-mediated psoriasis-like inflammation was suppressed in B cell-specific PTEN-deficient mice along with decreased ear thickness and epidermal thickness on day 15. Moreover, adoptive transfer of B1 B cells suppressed IL-23-mediated psoriasis-like inflammation. rmIL-23-injected B cell-specific PTEN-deficient mice showed expanded regulatory T cells (Tregs) in the spleen and draining lymph nodes along with increased Bregs. Further, T helper (Th) 17 differentiation in the rmIL-23-injected ear was suppressed in B cell-specific PTEN-deficient mice. Overall, these results indicate that increased Bregs suppress IL-23-mediated psoriasis-like inflammation through Treg expansion and inhibition of Th17 differentiation. Thus, targeting Bregs may be a feasible treatment strategy for psoriasis.

## Introduction

Psoriasis is a chronic, immune‐mediated, inflammatory cutaneous disease that causes erythematous, well-demarcated, oval plaques, with adherent silvery scales. It affects approximately 2% of the general population^1^. Because of its cutaneous condition, psoriasis has a negative impact on the patients’ quality of life^2^. Based on histological findings such as hyperkeratosis and acanthosis, psoriasis was previously considered a disorder with aberrant keratinocyte proliferation and differentiation. However, successful treatment with cyclosporine, a general suppressor of T cells, indicated that psoriasis is a T cell-mediated autoimmune disorder^3^. Although its pathogenetic mechanisms remain unclear, recent studies demonstrate that the interleukin (IL)-23/T helper (Th) 17 signaling pathway plays a critical role in psoriasis development^4^. IL-23 produced by activated antigen-presenting cells (APC) including dendritic cells (DC) stimulates and promotes Th17 cell differentiation. Th17 cells produce IL-17A and IL-22, which are the main cytokines in the pathogenesis of psoriasis. Based on the immunologic pathways underlying its pathogenesis, biologics targeting various immune-associated cytokines have greatly improved psoriasis treatment. However, only the role of T cells has been focused in the pathogenesis of psoriasis. In contrast, rituximab, a B cell-depleting monoclonal antibody (mAb), has been reported to induce psoriasiform skin lesions in humans^5,6^. This finding suggests that B cells have a negative regulatory role in psoriasis development even though it is represented as T cell-mediated autoimmune disorder.

B cells are central to the humoral immune response and produce antibodies against antigens. B cells also have multiple functions essential for immune homeostasis such as antigen presentation, T cell activation, and cytokine production^7^. B cells are known to regulate immune responses both positively and negatively. Regulatory B cells (Bregs) that produce IL-10 are identified as negative regulators of immune responses^8–11^. Absence or loss of Bregs exacerbates disease symptoms in several murine models such as experimental autoimmune encephalomyelitis (EAE)^12,13^, chronic colitis^14^, collagen-induced arthritis (CIA)^15^, contact hypersensitivity (CHS)^16,17^, lupus^18,19^, scleroderma^20^, and allergies^21^. Furthermore, Bregs are also reported to play an important role in various autoimmune diseases in humans^22,23^. Because of its essential role in Breg-mediated suppression, IL-10 production is a major identification marker for Bregs^24^. A recent study revealed that the phosphatidylinositol 3-kinase (PI3K)-Akt pathway, an important downstream effector of B-cell antigen receptor (BCR) signaling, promotes IL-10 production in B cells^25^. Deletion of the phosphatase and tensin homolog (PTEN), an inhibitor of Akt activity, results in hyper activation of Akt and promotes IL-10 production. We previously confirmed that PTEN inactivation specifically in B cells, using the Cre-loxP system results in Breg expansion. Bregs were significantly increased in the peripheral blood, spleen, and peritoneum of B cell-specific PTEN-deficient mice (*Cd19Cre*^+*/−*^*Pten*^*loxP/loxP*^ mice)^25^. Multiple Breg subpopulations have been described in mice. Mouse splenic Bregs are reported to exist within the CD1d^hi^CD5^+^ marginal zone (MZ) B cell subset^17^. However, another study showed that mouse splenic Bregs were found within the MZ as well as the B1 B cell subsets of B cell-specific PTEN-deficient mice^25^.

The role of Bregs in psoriasis remains poorly understood. However, Apremilast, a phosphodiesterase-4 (PDE4) inhibitor, was recently found to exerts its therapeutic effects in psoriasis through Breg expansion^26^. Clarifying the role of Bregs in psoriasis can thus contribute to the development of its treatment. Therefore, in this study, we assessed the role of Bregs in a model of IL-23-mediated psoriasis in B cell-specific PTEN-deficient mice.

## Results

### IL-23-mediated psoriasis-like inflammation was attenuated in B cell-specific PTEN-deficient mice

**Figure 1:**
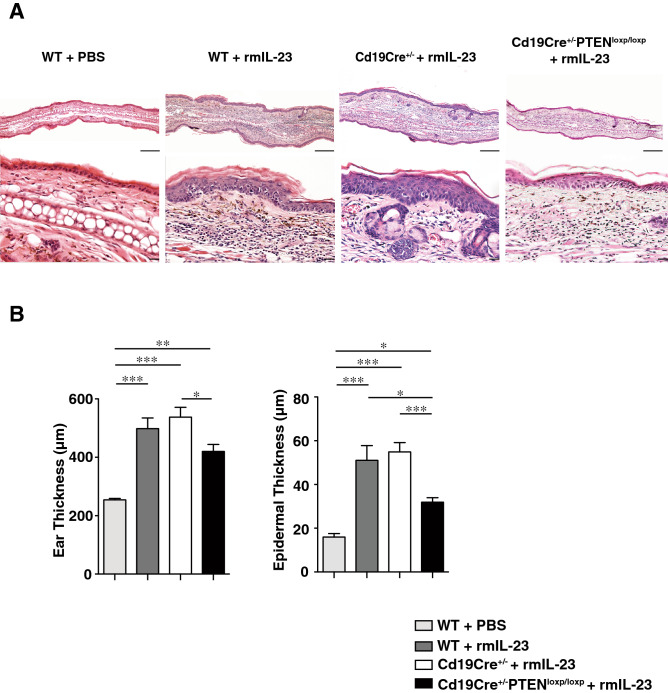
IL-23-mediated psoriasis-like inflammation was suppressed more in B cell-specific PTEN-deficient mice. (**A**) H&E staining of skin section from rmIL-23 or PBS-injected ears in WT mice, *Cd19Cre*^+*/−*^ mice and *Cd19Cre*^+*/−*^*Pten*^*loxP/loxP*^ mice. rmIL-23 induced hyperkeratosis, parakeratosis, acanthosis, and infiltration of mononuclear cells in the dermis (original magnification × 40, 200; bars = 200 μm and 20 μm). (**B**) Ear thickness and epidermal thickness were measured on day 15 in WT mice, *Cd19Cre*^+*/−*^ or *Cd19Cre*^+*/−*^*Pten*^*loxP/loxP*^ mice with intradermal injection of 20 μL PBS/0.1% BSA with or without 0.5 μg rmIL-23. Values represent means ± SEMs. Significant differences between sample means are indicated as: *P < 0.05, **P < 0.01, ***P < 0.001.

We treated wild type (WT) mice, *Cd19Cre*^+*/−*^ mice and *Cd19Cre*^+*/−*^*Pten*^*loxP/loxP*^ mice with intradermal injection of 20 μL PBS/0.1% BSA with or without 0.5 μg rmIL-23, into the right ear, every other day for 16 days. WT mice and *Cd19Cre*^+*/−*^ mice corresponded to the control in *Cd19Cre*^+*/−*^*Pten*^*loxP/loxP*^ mice, in which Bregs were significantly increased. Histopathologic assessment of rmIL-23-injected ears showed that rmIL-23 induced hyperkeratosis, parakeratosis, acanthosis, and mononuclear cell infiltration in the dermis. *Cd19Cre*^+*/−*^*Pten*^*loxP/loxP*^ mice had less marked hyperkeratosis and acanthosis compared to those in WT mice and *Cd19Cre*^+*/−*^ mice (Fig. 1A). rmIL-23 injection significantly increased ear thickness and epidermal thickness when compared with PBS control (*P* < 0.05, *P* < 0.01 and *P* < 0.001; Fig. 1B). Ear thickness on day 15 was greater in *Cd19Cre*^+*/−*^ mice compared to *Cd19Cre*^+*/−*^*Pten*^*loxP/loxP*^ mice (*P* < 0.05; Fig. 1B). Epidermal thickness on day 15 was also greater in WT mice and *Cd19Cre*^+*/−*^ mice compared to *Cd19Cre*^+*/−*^*Pten*^*loxP/loxP*^ mice (*P* < 0.05 and *P* < 0.001; Fig. 1B). These findings suggest that IL-23-mediated psoriasis-like inflammation was suppressed in *Cd19Cre*^+*/−*^*Pten*^*loxP/loxP*^ mice compared to control mice.

### Cell infiltration of rmIL-23-injected ears was decreased in B cell-specific PTEN-deficient mice

**Figure 2:**
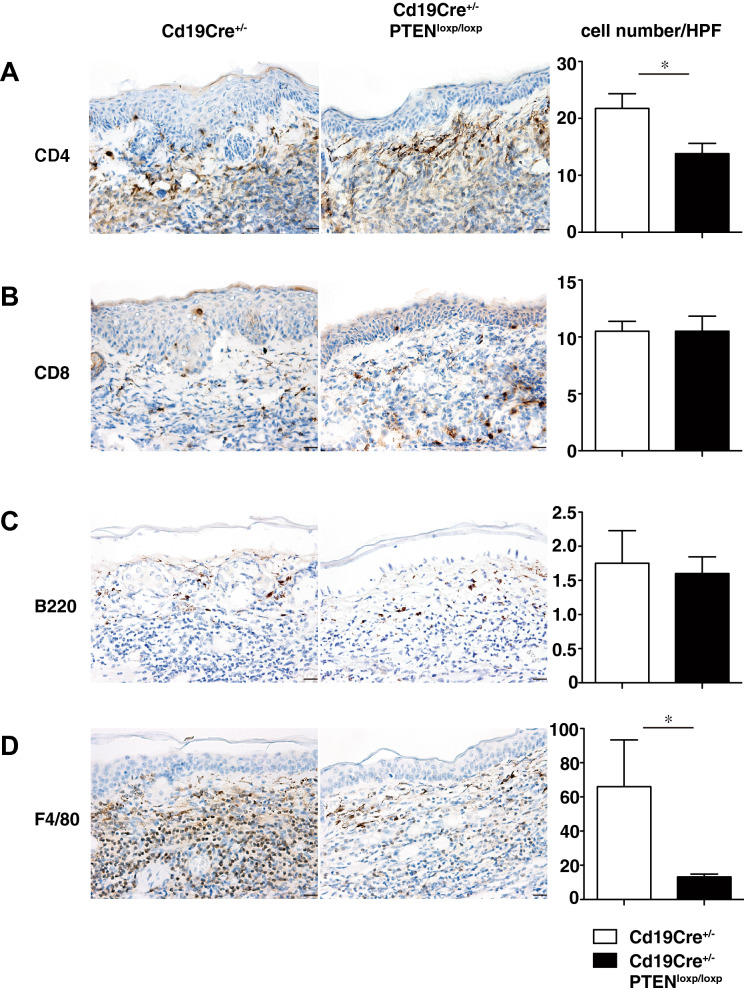
Cell infiltration of rmIL-23-injected ears was decreased in B cell-specific PTEN-deficient mice. Immunohistochemical staining of skin section from rmIL-23-injected ears in *Cd19Cre*^+*/−*^ and *Cd19Cre*^+*/−*^*Pten*^*loxP/loxP*^ mice (original magnification × 400; bars = 20 μm). The number of CD4^+^ T cells (**A**), CD8^+^ T cells (**B**), B cells (**C**) and F4/80^+^ macrophages (**D**) per field of view (× 400) were counted. Values represent means ± SEMs. Significant differences between samples means are indicated as: *P < 0.05, ***P < 0.001.

Immunohistochemical (IHC) staining of cellular infiltration in rmIL-23-injected ears showed decreased CD4^+^ T cells and F4/80^+^ macrophages in *Cd19Cre*^+*/−*^*Pten*^*loxP/loxP*^ mice compared to those in *Cd19Cre*^+*/−*^ mice (*P* < 0.05; Fig. 2A, D). The number of CD8^+^ T cells was not significantly different between both groups (Fig. 2B). There were a few B cells in the rmIL-23-injected ears both in *Cd19Cre*^+*/−*^ and *Cd19Cre*^+*/−*^*Pten*^*loxP/loxP*^ mice, but there were no significant differences between these groups (Fig. 2C). Thus, increased Bregs appear to suppress CD4^+^ T cell and F4/80^+^ macrophage infiltration in rmIL-23-injected ears.

### rmIL-23 injection increased the number of CD4^+^ T cells in the spleen and draining lymph nodes and B cells in the draining lymph nodes in B cell-specific PTEN-deficient mice

**Figure 3:**
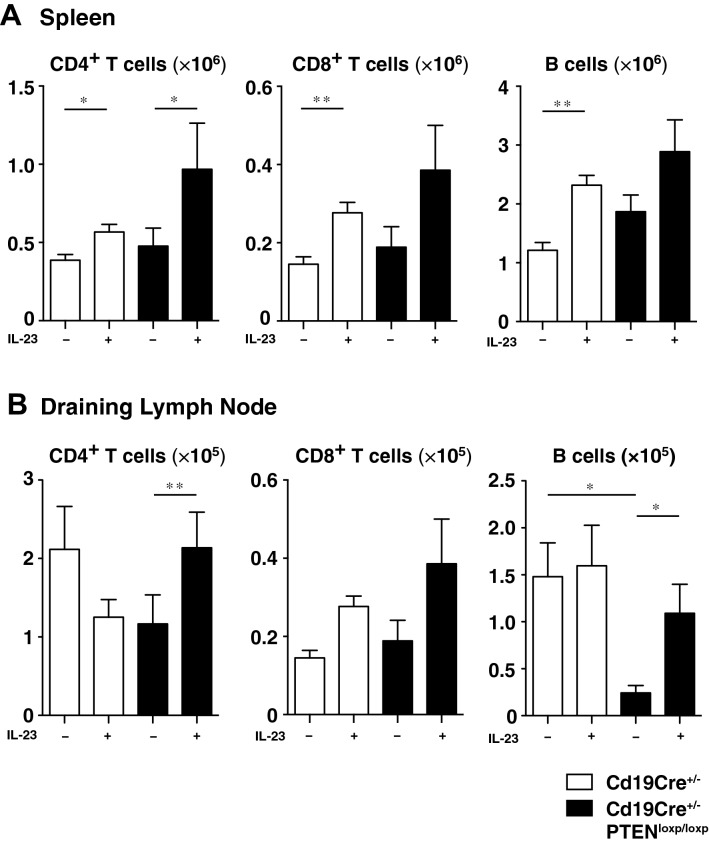
rmIL-23 injection increased the number of CD4^+^ T cells in the spleen and draining lymph nodes and B cells in the draining lymph nodes in B cell-specific PTEN-deficient mice. The number of CD4^+^ T cells, CD8^+^ T cells, and B cells in the spleen (**A**) and draining lymph nodes (**B**) of rmIL-23 injected *Cd19Cre*^+*/−*^ and *Cd19Cre*^+*/−*^*Pten*^*loxP/loxP*^ mice. Values represent means ± SEMs. Significant differences between samples means are indicated as: *P < 0.05, **P < 0.01.

To determine whether rmIL-23 injection affected the composition of CD4^+^ T cells, CD8^+^ T cells, and B cells, we prepared single-cell suspensions from the spleen and draining lymph nodes of *Cd19Cre*^+*/−*^and *Cd19Cre*^+*/−*^*Pten*^*loxP/loxP*^ mice before injection on day 0 and on day 15 and analyzed them by flow cytometry. rmIL-23 injection significantly increased the number of CD4^+^ T cells in the spleen in both groups (*P* < 0.05; Fig. 3A). rmIL-23 injection significantly increased the number of CD8^+^ T cells and B cells in the spleen of *Cd19Cre*^+*/−*^ mice (*P* < 0.01; Fig. 3A), whereas increasement of CD8^+^ T cells and B cells in rmIL-23-injected *Cd19Cre*^+*/−*^*Pten*^*loxP/loxP*^ mice was not significant. rmIL-23 injection significantly increased the number of CD4^+^ T cells and B cells in the draining lymph nodes of *Cd19Cre*^+*/−*^*Pten*^*loxP/loxP*^ mice (*P* < 0.05 and *P* < 0.01; Fig. 3B). The number of B cells before rmIL-23 injection in the draining lymph nodes was decreased in *Cd19Cre*^+*/−*^*Pten*^*loxP/loxP*^ mice compared with those in *Cd19Cre*^+*/−*^ mice (*P* < 0.05; Fig. 3B). Thus, rmIL-23 injection increased the number of CD4^+^ T cells in the spleen in both groups, CD8^+^ T cells and B cells in the spleen of *Cd19Cre*^+*/−*^ mice and CD4^+^ T cells and B cells in the draining lymph nodes of *Cd19Cre*^+*/−*^*Pten*^*loxP/loxP*^ mice.

### Tregs in the spleen and draining lymph nodes were expanded in rmIL-23-injected B cell-specific PTEN-deficient mice along with increased Bregs

**Figure 4:**
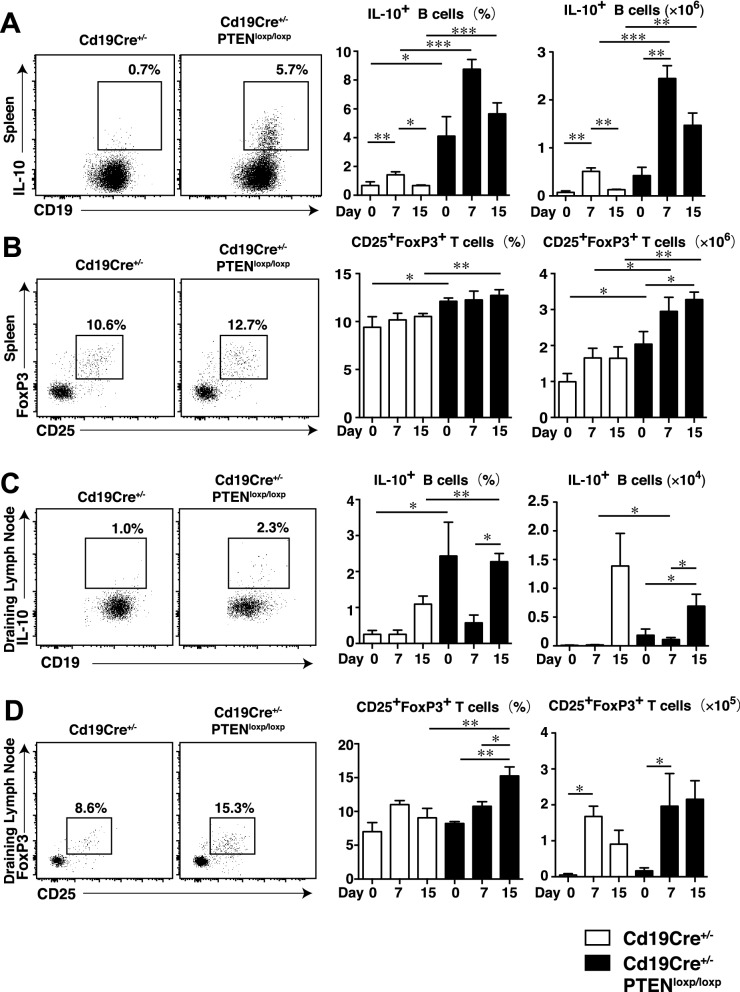
Tregs in the spleen and draining lymph nodes were expanded in rmIL-23-injected B cell-specific PTEN-deficient mice along with increased Bregs. The frequencies and numbers of IL-10-producing B cells (**A**) and CD25^+^FoxP3^+^ T cells (**B**) in the spleen of rmIL-23-injected *Cd19Cre*^+*/−*^ and *Cd19Cre*^+*/−*^*Pten*^*loxP/loxP*^ mice. The frequencies and numbers of IL-10-producing B cells (**C**) and CD25 + FoxP3 + T cells (**D**) in the draining lymph node of rmIL-23-injected *Cd19Cre*^+*/−*^ and *Cd19Cre*^+*/−*^*Pten*^*loxP/loxP*^ mice. The images were created with FlowJo software (version 10.1R5; Tree Star, San Carlos, CA, USA, https://www.flowjo.com/). Significant differences between samples means are indicated as: *P < 0.05, **P < 0.01, ***P < 0.001.

We next analyzed the frequencies of Bregs and Tregs in the spleen and draining lymph nodes of *Cd19Cre*^+*/−*^ and *Cd19Cre*^+*/−*^*Pten*^*loxP/loxP*^ mice on days 0, 7, and 15 and analyzed them by flow cytometry. In the spleen, the frequencies of IL-10-producing B cells was significantly increased in *Cd19Cre*^+*/−*^*Pten*^*loxP/loxP*^ mice compared to those in *Cd19Cre*^+*/−*^ mice during all courses of IL-23-mediated psoriasis-like inflammation (*P* < 0.05 and *P* < 0.001; Fig. 4A). rmIL-23 injection significantly increased the number of IL-10-producing B cells in the spleen in both groups on day 7, while the number of IL-10-producing B cells was significantly increased in *Cd19Cre*^+*/−*^*Pten*^*loxP/loxP*^ mice compared to those in *Cd19Cre*^+*/−*^ mice on days 7 and 15 (*P* < 0.01 and *P* < 0.001; Fig. 4A). In the spleen, the frequencies of CD25^hi^ FoxP3^+^ CD4^+^ Tregs was significantly increased in *Cd19Cre*^+*/−*^*Pten*^*loxP/loxP*^ mice compared to those in *Cd19Cre*^+*/−*^ mice on days 0 and 15 (*P* < 0.05 and *P* < 0.01; Fig. 4B). The number of CD25^hi^ FoxP3^+^ CD4^+^ Tregs in the spleen was significantly increased in *Cd19Cre*^+*/−*^*Pten*^*loxP/loxP*^ mice compared to those in *Cd19Cre*^+*/−*^ mice during all courses of IL-23-mediated psoriasis-like inflammation and increased gradually relative to Bregs during the course of disease progression in *Cd19Cre*^+*/−*^*Pten*^*loxP/loxP*^ mice (*P* < 0.05 and *P* < 0.01; Fig. 4B). In the draining lymph nodes, the frequencies of IL-10-producing B cells was significantly increased in *Cd19Cre*^+*/−*^*Pten*^*loxP/loxP*^ mice compared to those in *Cd19Cre*^+*/−*^ mice on days 0 and 15 (*P* < 0.05 and *P* < 0.01; Fig. 4C). rmIL-23 injection significantly increased the number of IL-10-producing B cells in the draining lymph nodes of *Cd19Cre*^+*/−*^*Pten*^*loxP/loxP*^ mice (*P* < 0.05; Fig. 4C). rmIL-23 injection significantly increased the frequencies of CD25^hi^ FoxP3^+^ CD4^+^ Tregs in the draining lymph nodes during the course of disease progression in *Cd19Cre*^+*/−*^*Pten*^*loxP/loxP*^ mice (*P* < 0.05 and *P* < 0.01; Fig. 4D). The frequencies of CD25^hi^ FoxP3^+^ CD4^+^ Tregs in the draining lymph nodes was also significantly increased in *Cd19Cre*^+*/−*^*Pten*^*loxP/loxP*^ mice compared to those in *Cd19Cre*^+*/−*^ mice on day15 (*P* < 0.01; Fig. 4D). rmIL-23 injection significantly increased the number of CD25^hi^ FoxP3^+^ CD4^+^ Tregs in the draining lymph nodes in both groups on day 7 (*P* < 0.05; Fig. 4D). Our data thus suggests that Bregs in the spleen were significantly expanded in rmIL-23-injected B cell-specific PTEN-deficient mice and expanded significantly on day 7 and contracted on day15 while keeping high levels. On the other hands, Tregs in the spleen and draining lymph nodes were significantly expanded in rmIL-23-injected B cell-specific PTEN-deficient mice and increased gradually during late-phase of IL-23-mediated psoriasis-like inflammation along with increased Bregs.

### Th17 differentiation in rmIL-23-injected ears was suppressed in B cell-specific PTEN-deficient mice

**Figure 5:**
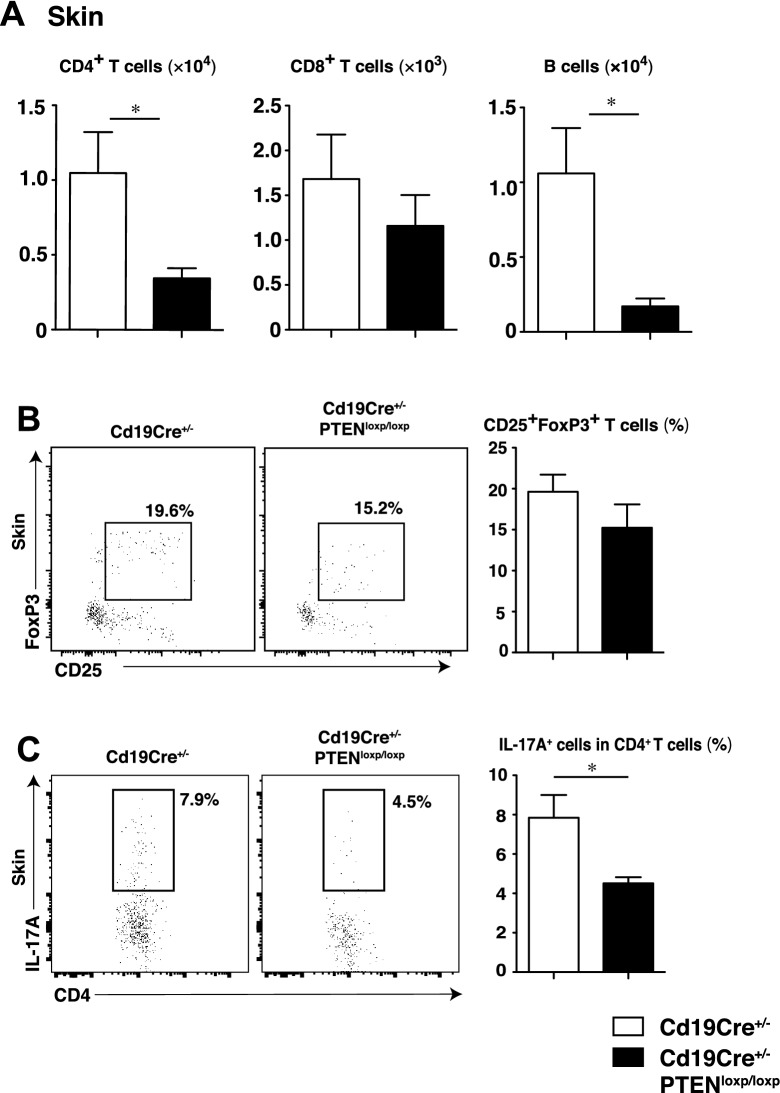
Th17 differentiation in rmIL-23-injected ears was suppressed in B cell-specific PTEN-deficient mice. The number of CD4^+^ T cells, CD8^+^ T cells, and B cells in the inflamed skin of rmIL-23 injected *Cd19Cre*^+*/−*^ and *Cd19Cre*^+*/−*^*Pten*^*loxP/loxP*^ mice (**A**). The frequencies of CD25^+^FoxP3^+^ T cells (**B**) and CD4^+^IL-17A^+^ T cells in the inflamed skin of rmIL-23-injected *Cd19Cre*^+*/−*^ and *Cd19Cre*^+*/−*^*Pten*^*loxP/loxP*^ mice (**C**). The images were created with FlowJo software (version 10.1R5; Tree Star, San Carlos, CA, USA, https://www.flowjo.com/).

Th17 cells, which are stimulated by IL-23, play a central role in psoriasis development through IL-17A and IL-22 production. IL-17A is reported as necessary to produce IL-23-mediated psoriasis-like inflammation^27^. To assess whether increased Bregs suppress Th17 cell differentiation, the composition of CD4^+^ T cell, CD8^+^ T cells, B cells, Tregs, and IL-17A producing CD4^+^ T cells in rmIL-23-injected ears was evaluated by flow cytometry. The numbers of CD4^+^ T cells and B cells in rmIL-23-injected ears were significantly decreased in rmIL-23-injected *Cd19Cre*^+*/−*^*Pten*^*loxP/loxP*^ mice compared with that in *Cd19Cre*^+*/−*^ mice (Fig. 5A). The frequencies of CD25^hi^ FoxP3^+^ CD4^+^ Tregs in rmIL-23-injected ears was not significantly different between both groups (Fig. 5B). The frequencies of IL-17A producing CD4^+^ T cells in rmIL-23-injected ears was significantly decreased in rmIL-23-injected　*Cd19Cre*^+*/−*^*Pten*^*loxP/loxP*^ mice when compared with those in *Cd19Cre*^+*/−*^ mice (Fig. 5C). Breg infiltration was not observed in the ears during IL-23-mediated psoriasis-like inflammation. Our data suggest that increased Bregs in the spleen and draining lymph nodes suppress Th17 cell differentiation in rmIL-23-injected ears.

### Adoptive transfer of B1 B cells suppressed IL-23-mediated psoriasis-like inflammation

**Figure 6:**
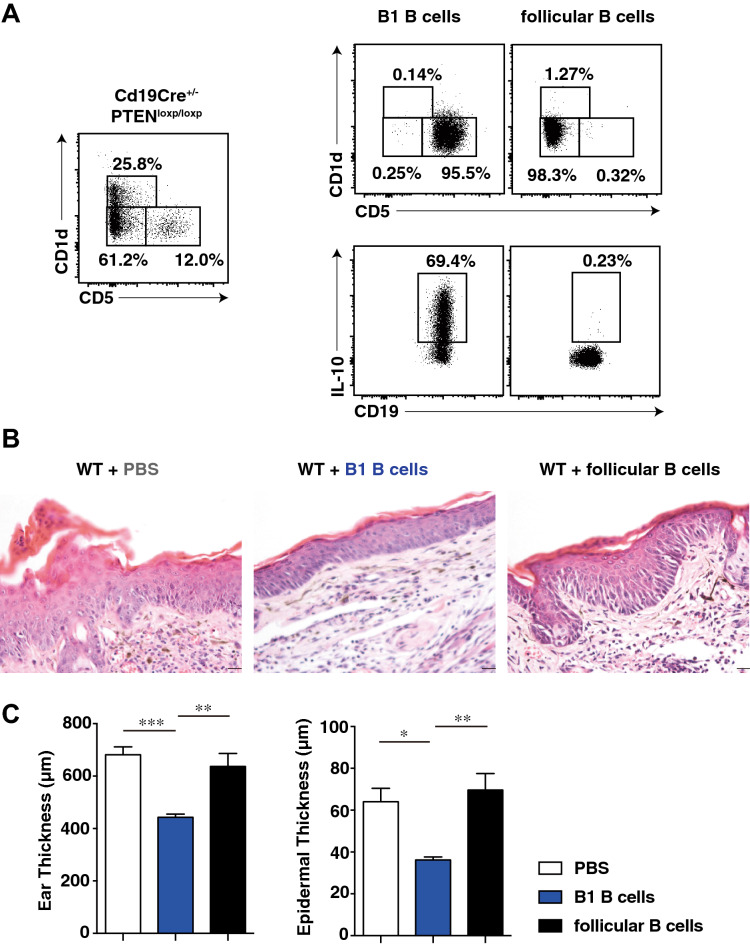
Adoptive transfer of B1 B cells suppressed IL-23-mediated psoriasis-like inflammation. (**A**) Splenic B cells from *Cd19Cre*^+*/−*^*Pten*^*loxP/loxP*^ mice were isolated, stained for CD1d, CD5, and CD19 expression; and sorted into CD1d^int^CD5^+^ B1 B cells and CD1d^int^CD5^-^ follicular B cells before stimulation with a mixture of LPS, PMA, ionomycin, and brefeldin-A for 5 h. IL-10^+^ B cells derived from each purified population were analyzed using flow cytometry. (**B**) Isolated splenic B cells (1 × 10^6^ cells) from *Cd19Cre*^+*/−*^*Pten*^*loxP/loxP*^ mice were transferred into WT mice before rmIL-23 injection. H&E staining of skin section from ears in WT mice (original magnification × 20; bars = 20 μm). (**C**) Effect of adoptive transfer of B1 B cells on ear thickness and epidermal thickness. The images were created with FlowJo software (version 10.1R5; Tree Star, San Carlos, CA, USA, https://www.flowjo.com/). Values represent means ± SEMs (n = 10 mice/group). Significant differences between samples means are indicated as: *P < 0.05, **P < 0.01, ***P < 0.001.

We next evaluated whether Bregs could suppress IL-23-mediated psoriasis-like inflammation by adoptive transfer experiments. Mouse splenic Bregs have been predominantly found within the B1 B cell subset in *Cd19Cre*^+*/−*^*Pten*^*loxP/loxP*^ mice^25^. Thus, splenic B cells from *Cd19Cre*^+*/−*^*Pten*^*loxP/loxP*^ mice were isolated and stained for CD1d, CD5, and CD19 expression and were then sorted into 3 subsets, CD1d^int^CD5^+^ B1 B cells, CD1d^hi^CD5^+^ MZ B cells, and CD1d^int^CD5^-^ follicular B cells. B1 B cells and follicular B cells were stimulated in vitro with a mixture of LPS, PMA, ionomycin, and brefeldin-A for 5 h. As reported previously, B1 B cells accounted for more than 60% of IL-10^+^ B cells, whereas follicular B cells accounted for less than 1% of IL-10^+^ B cells (Fig. 6A). Next, isolated B1 B cells, follicular B cells and PBS control were adoptively transferred into WT mice before rmIL-23 injection. Adoptive transfer of follicular B cells and PBS control had no effect on hyperkeratosis, parakeratosis, and acanthosis induced by IL-23 whereas adoptive transfer of B1 B cells reduced IL-23-mediated psoriasis-like inflammation (Fig. 6B). Adoptive transfer of B1 B cells significantly reduced ear thickness and epidermal thickness on day 15 when compared with the adoptive transfer of follicular B cells and PBS control (*P* < 0.05, *P* < 0.01 and *P* < 0.001; Fig. 6C). Thus, adoptive transfer of B1 B cells directly influenced the suppression of IL-23-mediated psoriasis-like inflammation. Furthermore, we evaluated how Bregs could suppress IL-23-mediated psoriasis-like inflammation by using a function-blocking mAb against the IL-10 receptor. Blocking IL-10 receptor function tended to increase ear thickness in rmIL-23-injected *Cd19Cre*^+*/−*^*Pten*^*loxP/loxP*^ mice compared to control mice; however, the difference was not statistically significant (Supplementary Fig. 1). Thus, it is possible that suppression of IL-23-mediated psoriasis-like inflammation by Bregs was mainly IL-10-dependent.

## Discussion

To our knowledge, this study is the first to evaluate the role of Bregs in IL-23-mediated psoriasis-like inflammation. Histopathologic assessment of rmIL-23-injected ears showed that *Cd19Cre*^+*/−*^*Pten*^*loxP/loxP*^ mice had less marked hyperkeratosis and acanthosis compared to *Cd19Cre*^+*/−*^ control mice. Moreover, adoptive transfer of B1 B cells directly suppressed the symptoms of IL-23-mediated psoriasis-like inflammation. IHC of cellular infiltration in rmIL-23-injected ears showed decreased CD4^+^ T cells and F4/80^+^ macrophages in *Cd19Cre*^+*/−*^*Pten*^*loxP/loxP*^ mice. Tregs were expanded in the spleen and draining lymph nodes of rmIL-23-injected *Cd19Cre*^+*/−*^*Pten*^*loxP/loxP*^ mice in association with increased Bregs. Further, Th17 differentiation in rmIL-23-injected ears was suppressed in *Cd19Cre*^+*/−*^*Pten*^*loxP/loxP*^ mice. Overall, these results indicate that increased Bregs suppress IL-23-mediated psoriasis-like inflammation through Treg expansion and inhibition of Th17 differentiation. Thus, targeting Bregs may be a feasible treatment strategy for psoriasis.

A previous study has shown that IL-23 gene expression is elevated in human psoriatic skin lesions^28^. In addition, direct administration of IL-23 into the skin of mice induces psoriasis-like inflammation^29–31^. Although histological findings with rmIL-23 injection were reported to be maximal at day 4^29^, Hedrick et al. reported that prominent intracorneal pustules early on day 5 progress to increasing acanthosis and dermal inflammation on day 15. This temporal progression of histological features suggests progression from a T cell-independent to a T cell-dependent phase of the rmIL-23-induced response^32^. In the present study, rmIL-23 injection induced psoriasis-like inflammation with remarkable acanthosis and dermal inflammation on day 15. The current study showed that IL-23-mediated psoriasis-like inflammation was more suppressed in *Cd19Cre*^+*/−*^*Pten*^*loxP/loxP*^ mice compared with that in *Cd19Cre*^+*/−*^ mice, suggesting that increased Bregs inhibit IL-23-mediated psoriasis-like inflammation. Furthermore, adoptively transferred B1 B cells directly suppressed the symptoms of IL-23-mediated psoriasis-like inflammation. Thus, Bregs play a critical role in suppressing IL-23-mediated psoriasis-like inflammation. In a mouse model of imiquimod-induced psoriasis-like inflammation, Bregs also play an important role. Yanaba et al. have reported that CD19-deficient mice which have few Bregs increased severity of imiquimod-induced psoriasis-like inflammation^33^. Alrefai et al. have also reported that imiquimod-induced psoriasis-like inflammation was exacerbated in mice depleted of B cells or bearing IL-10 deficient B cells^34^. These findings suggest that Bregs exert their suppressive function in different mouse model of psoriasis. Consistent with this finding, treatment with rituximab, a B cell-depleting mAb, has been reported to induce psoriasiform skin lesions in human subjects without a previous history of psoriasis^5,6^. Psoriasis is an immune-mediated disease where T cells play a central role in establishing inflammation in psoriatic skin lesions; indeed, numerous biological therapies approved for treating psoriasis target either T cells or cytokines. Our results suggest that Bregs inhibit IL-23-mediated psoriasis-like inflammation by suppressing T cell responses.

There are multiple mechanisms by which Bregs exert their negative regulatory function on immune responses. Bregs regulate T cell responses through inhibition of Th1 and Th17 cell differentiation while promoting Treg expansion^35^. As Tregs play a critical role in regulating immune tolerance and preventing autoimmune diseases, defects in Treg function or reduced numbers of Tregs have been reported in several autoimmune diseases. Similarly, the suppressor function of Tregs is reported to be deficient in psoriasis^36^. Moreover, several psoriasis treatments increase the number of Tregs or restore their function for clinical improvement in humans. Previous studies have suggested that Bregs predominantly regulate the early phase disease, whereas Tregs reciprocally attenuate late-phase disease^17,37^. Our current study indicates that Tregs in the spleen and draining lymph nodes were significantly expanded in rmIL-23-injected B cell-specific PTEN-deficient mice on day 15 and increased gradually during late-phase of IL-23-mediated psoriasis-like inflammation in association with increased Bregs. IL-23-induced inflammation progresses from a T cell-independent to a T cell-dependent phase. These findings suggest that Bregs suppress IL-23-induced psoriasis-like inflammation through Treg expansion in the late-phase disease. The current study also showed that adoptive transfer of B1 B cells before starting rmIL-23 injection significantly reduced the severity of inflammation, suggesting that Bregs play an important role in triggering the onset of psoriasis. Therefore, this result suggests that Bregs play a major role in suppressing IL-23-mediated psoriasis-like inflammation in the early phase, whereas Tregs expanded by Bregs inhibit the late-phase condition. Our current results also demonstrate that Th17 differentiation in rmIL-23-injected ears was suppressed in *Cd19Cre*^+*/−*^*Pten*^*loxP/loxP*^ mice, suggesting that Bregs inhibit IL-17A producing CD4^+^ T cells in rmIL-23-injected ears. Taken together, Bregs have the ability to shift CD4 differentiation from Th17 cells toward Tregs. A previous study has shown that human CD19^+^CD24^hi^CD38^hi^ B cells inhibited naïve T cell differentiation into Th1 and Th17 cells and converted CD4^+^ CD25^−^T cells into Tregs, in part through the production of IL-10^38^. In this study, blocking IL-10 receptor function tended to increase ear thickness in rmIL-23-injected *Cd19Cre*^+*/−*^*Pten*^*loxP/loxP*^ mice compared to control mice; however, the difference was not statistically significant. It is possible that suppression of IL-23-mediated psoriasis-like inflammation by Bregs was mainly IL-10-dependent.

Our current study showed that Bregs in the spleen and draining lymph nodes were significantly increased in *Cd19Cre*^+*/−*^*Pten*^*loxP/loxP*^ mice, while we did not observe directly the migration of Bregs into inflamed skin. These results suggest that Bregs exert their negative regulatory function by producing IL-10 in lymphoid tissues such as the spleen and draining lymph nodes. However, we cannot exclude the possibility that Bregs directly suppress IL-23-induced inflammation at inflamed skin. Further studies will be needed to confirm the migration of Bregs into inflames skin.

In conclusion, our results demonstrate the critical role of Bregs in psoriasis in mice.

Rituximab treatment has been reported to induce psoriasiform skin lesions in humans^5,6^. On the other hands, there is also a report of successful treatment of psoriatic arthritis with rituximab in humans^39^. This discrepancy suggests that the role of B cells in psoriasis is complex in humans. However, recent studies have demonstrated that Bregs progenitor cells were significantly increased, while Bregs were significantly decreased in the peripheral blood of patients with psoriasis and pustular psoriasis^40,41^. These results indicate that Bregs progenitor cells increased in response to decreasing Bregs as a negative feedback mechanism and Bregs were functionally impaired in patients with psoriasis and pustular psoriasis. There is now accumulating evidence that Bregs are crucial in pathogenesis of psoriasis not only in mice but also in humans. Our study reveals that Bregs suppress IL-23-mediated psoriasis-like inflammation through Treg expansion and inhibition of Th17 differentiation. Thus, Breg-targeting may provide a feasible therapeutic strategy in psoriasis and further studies are required to develop such approaches.

## Materials and methods

### Animals

Female C57BL/6 mice, *Cd19Cre*^+*/−*^ mice, and *Pten*^*loxP/loxP*^ mice of the C57BL/6 background were obtained from the Jackson Laboratory (Bar Harbor, Me, USA). All mice were housed in a specific pathogen-free barrier facility with ad libitum access to food and water and maintained on 12 h-light/12 h-dark light cycle with the lights on at 8:45 AM. All mice used were 8 to 12 weeks of age. All studies and procedures were approved by the Committee on Animal Experimentation of Kanazawa University Graduate School of Medical Science. All animal experiments were conducted in accordance with the ARRIVE guidelines (Animal Research: Reporting of In Vivo Experiments) and the ethical guidelines of Kanazawa University. All efforts were made to minimize the number of animals used and pain suffering to mice.

### Intradermal cytokine injection

Mice were injected intradermally with 20 μL PBS/0.1% bovine serum albumin (BSA) containing 0.5 μg recombinant mouse (rm)IL-23 (BioLegend, San Diego, CA, USA) in the right ear using a 29-gauge needle, every other day for 16 days^32^. Ear thickness was measured in a blinded manner with a micrometer before injection on days 0 and 15. All mice were euthanized using carbon dioxide asphyxiation on days 0, 7, and 15 and samples were collected for evaluation. For the Ab blocking experiments, mice were intraperitoneally injected with IL-10 receptor (1B1.3a clone; BioLegend) or control monoclonal antibodies (mAbs) (250 μg) 1 h before injections into ear on day 0, 4, 8 and 12.

### Histologic examination and immunohistochemical staining

Ear samples were fixed in 10% formalin and embedded in paraffin. Sections were stained with hematoxylin and eosin (H&E). As we described previously in our study^42^, for immunohistochemical (IHC) staining of CD4 and CD8, the sections were frozen in Tissue-Tek OCT compound (Sakura Finetek, Tokyo, Japan) and quickly frozen in liquid nitrogen. Frozen sections were fixed in cold acetone for 5 min and incubated with rat mAbs specific for mouse CD4 (RM4–5 clone; BD Biosciences, San Jose, CA, USA) and CD8 (53–6.7 clone; BD Biosciences). For IHC staining of B220 and F4/80, the slides were deparaffinized in xylene and 3% H_2_O_2_, hydrated through graded alcohols, and washed in distilled water. The slides were then placed in protease K (S3020; DAKO, Santa Clara, CA, USA) for 20 min and then blocked by incubating the slides for 30 min in normal goat serum with Tris-buffered saline (TBS). The sections were incubated with antibodies against B220 (RA3-6B2 clone; BD Biosciences) and macrophages (F4/80, BM8 clone; BioLegend). The sections were then incubated sequentially (20 min at 37℃) with a biotinylated goat anti-rabbit IgG secondary antibody (BD Biosciences) and then with a horseradish peroxidase-conjugated avidin–biotin complex (Vectastain ABC; Vector Laboratories, Burlingame, CA, USA). All sections were washed 3 times with PBS between incubations, developed with 3,3ʹ-diaminobenzidine tetrahydrochloride, and H_2_O_2_, and then counterstained with hematoxylin. Sections from at least four mice per group were stained and photomicrographs of representative fields were taken at × 400 magnification. Average numbers of positive cells per photomicrograph were counted. Each section was examined independently by two investigators in a blinded manner.

### Surface and intracellular staining for flow cytometry

#### Cell surface staining

Single-cell leukocyte suspensions from the spleen and draining lymph nodes were generated by gentle dissection. The following mAbs were used for staining: anti CD4-PE (L3T4 GK1.5), and anti-B220-Fluorescein isothiocyanate (FITC; RA3-6B2) from BD Biosciences; anti CD8a-Pacific Blue (53–6.7) from eBioscience (Waltham, MA, USA); anti-CD1d-FITC (1B1), anti-CD5-PE (53–7.3), anti-CD11b-PerCP/Cy5.5 (M1/70), anti-CD19-APC (6D5), anti-CD19-PE-Cy7 (6D5), anti-CD90.2-PE/Cy7 (30-H12), anti-F4/80-APC/Cy7 (BM8), anti-IL-10-PE (JES5-16E3), anti-IL-10-APC (JES5-16E3), and anti-IL-17A-APC (TC11-18H10.1) from BioLegend; and FoxP3-PE (FJK-16 s) from Invitrogen (Carlsbad, CA, USA). LIVE/DEAD Fixable Aqua Dead Cell Stain (Invitrogen) was used to detect dead cells. Single-cell suspensions (10^6^ cells) from the spleen and draining lymph nodes were stained at 4 °C for 20 min using mAbs at predetermined optimal concentrations.

#### Intracellular staining for FoxP3

For intracellular staining, cells were washed, fixed, and permeabilized for 30 min at 4 °C using the Foxp3/Transcription Factor Staining Buffer Set (eBioscience). Cells were then washed with permeabilization Buffer (eBioscience) and stained for FoxP3 at room temperature for 30 min.

#### Intracellular cytokine staining for IL-10 and IL-17

As we described previously in our study^20^, isolated leukocytes (2 × 10^6^ cells/mL) were resuspended in complete medium [RPMI 1640 medium-10% fetal bovine serum containing penicillin (200 μg/mL), streptomycin (200 U/mL), 4 mmol/L L-glutamine, and 5 × 10^−5^ mol/L 2-mercaptoethanol; all from Gibco, Carlsbad, CA, USA] and stimulated with lipopolysaccharide (LPS; 10 μg/mL; *Escherichia coli* serotype 0111: B4; Sigma-Aldrich, St louis, Mo, USA), phorbol 12 myristate 13-acetate (PMA; 50 ng/mL, Sigma-Aldrich), ionomycin (500 ng/mL, Sigma-Aldrich), and brefeldin A (3 μM, BioLegend) for IL-10 and PMA, ionomaycin, and brefeldin A for IL-17 for 5 h at 37℃. Dead cells were detected using a LIVE/DEAD Fixable Aqua Dead Cell Stain Kit (Invitrogen) before cell surface staining. Stained cells were fixed and permeabilized using a Cytofix/Cytoperm kit (BD Biosciences) according to the manufacturer’s instructions and stained with PE or APC -conjugated mouse anti-IL-10 mAb and APC-conjugated mouse anti-IL-17A mAb.

#### Flow cytometry

Stained samples were analyzed on a FACS Canto II flow cytometer (BD Biosciences). Data were analyzed with FlowJo software (version 10.1R5; Tree Star, San Carlos, CA, USA).

### Preparation of single-cell suspensions of ear-infiltrating lymphocytes for flow cytometry

As we described previously in our study^43^, the ear samples were minced on day 15 and enzymatically digested with 6.47 mL of RPMI 1640 medium-10% fetal bovine serum containing 2 mg/mL crude collagenase D (Roche, Basel, Switzerland), 1.5 mg/mL hyaluronidase (Sigma-Aldrich), and 0.03 mg/mL DNase I (Sigma-Aldrich) at 37 °C for 90 min to prepare the cell suspensions for flow cytometry. Digested cells were passed through a 70-μm cell Falcon Cell Strainer (BD Biosciences) to generate a single-cell suspension. The cell suspension was then centrifuged at 460 × *g* for 20 min. The pellet was resuspended in 70% Percoll solution (GE Healthcare, Uppsala, Sweden), and overlaid with a 37% Percoll solution followed by centrifugation at 500 × *g* for 20 min at room temperature. Cells were aspirated from the Percoll interface and passed through a 70-μm cell strainer. The cells were harvested by centrifugation and washed with PBS.

### Adoptive transfer of B cells

Splenic B cells from *Cd19Cre*^+*/−*^*Pten*^*loxP/loxP*^ mice were isolated by negative selection with anti-Thy1.2 microbeads (Miltenyi Biotec, Auburn, CA, USA) and stained for CD1d, CD5, and CD19 expression. Splenic B cells from *Cd19Cre*^+*/−*^*Pten*^*loxP/loxP*^ mice were sorted using FACS Aria Fusion (BD Bioscience) into 3 subsets: CD1d^int^CD5^+^ B1 B cells, CD1d^hi^CD5^+^ MZ B cells, and CD1d^int^CD5^-^ follicular B cells with 87–97% purity. Purified B1 B cells (1 × 10^6^ cells) and follicular B cells (1 × 10^6^ cells) were transferred into wild type mice by intravenous injection before injecting rmIL-23 on day 0.

### Statistical analysis

All data are shown as the mean ± standard error of the mean. The significance of differences between sample means was determined by Student’s *t* test. Bonferroni’s test was used for multiple comparisons. *P* values < 0.05 were considered statistically significant.

## Supplementary information

Supplementary Information.
