# Supplementary material for: Suppression of IL-23-mediated psoriasis-like inflammation by regulatory B cells
Source: Sci Rep. 2021 Jan 22;11:2106. doi: 10.1038/s41598-021-81588-8 (PMC7822829; doi:10.1038/s41598-021-81588-8)
Supplement: Supplementary file 1 — Supplementary Information. [file 41598_2021_81588_MOESM1_ESM.pdf]

Supplementary Information

Suppression of IL-23-Mediated Psoriasis-like Inflammation by Regulatory B cells

Kie Mizumaki[1], Motoki Horii[1], Miyu Kano[1], Akito Komuro,[1, 2] and Takashi Matsushita[1]

[1] Department of Dermatology, Faculty of Medicine, Institute of Medical, Pharmaceutical and Health Sciences, Kanazawa University, Kanazawa 920-8641, Japan

[2]Department of Plastic surgery, Kanazawa University Hospital, Kanazawa 920-8641, Japan

Address correspondence and reprint requests to: Takashi Matsushita MD, PhD, Department of Dermatology, Faculty of Medicine, Institute of Medical, Pharmaceutical and Health Sciences, Kanazawa University, Kanazawa 920-8641, Japan.

Phone: 81-76-265-2343

Fax: 81-76-234-4270

E-mail: t-matsushita@med.kanazawa-u.ac.jp

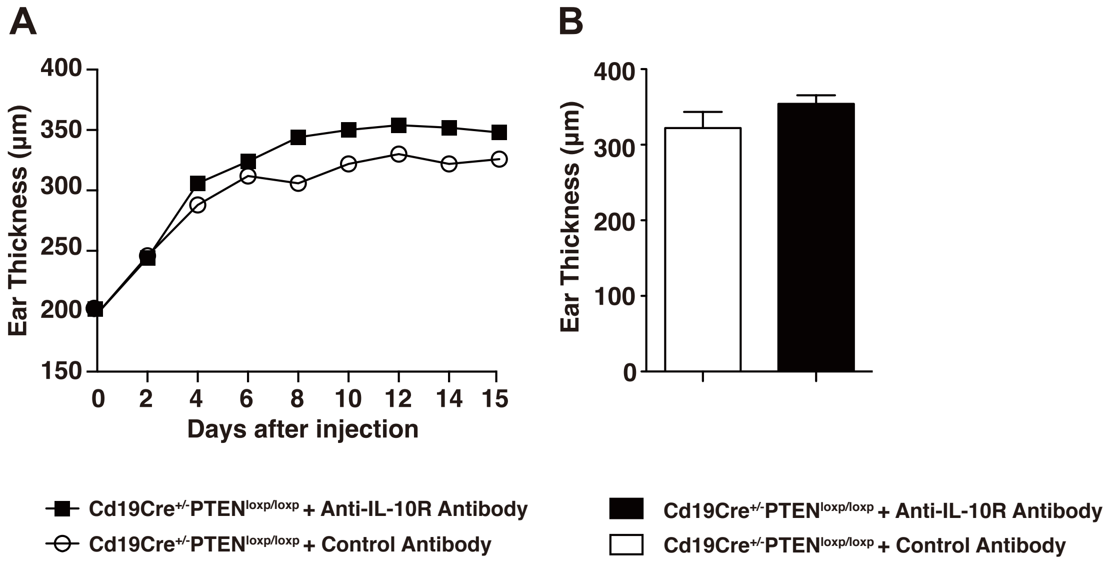

Supplementary Figure.

Cd19Cre$^{+/−}$*Pten*$^{loxP/loxP}$ mice were treated with IL-10 receptor mAb or control mAb 1 hour before injections into ear on day 0, 4, 8 and 12. Ear thickness was measured on days before injections (A) and Day 15 (B). Values represent means ± SEMs (n = 5 mice/group).
